# Vamorolone, a dissociative steroidal compound, reduces pro-inflammatory cytokine expression in glioma cells and increases activity and survival in a murine model of cortical tumor

**DOI:** 10.18632/oncotarget.14070

**Published:** 2016-12-21

**Authors:** Elizabeth Wells, Madhuri Kambhampati, Jesse M Damsker, Heather Gordish-Dressman, Sridevi Yadavilli, Oren J Becher, Jamila Gittens, Mojca Stampar, Roger J Packer, Javad Nazarian

**Affiliations:** ^1^ Research Center for Genetic Medicine, Children's National Health System, Washington, DC, USA; ^2^ Brain Tumor Institute, Center for Neuroscience and Behavioral Medicine, Children's National Health System, Washington, DC, USA; ^3^ ReveraGen BioPharma, Rockville, MD, USA; ^4^ Duke University Medical Center, Durham, NC, USA; ^5^ Department of Integrative Systems Biology, George Washington University School of Medicine and Health Sciences, Washington, DC, USA

**Keywords:** glucocorticoids, cytokines, anti-inflammatory, pre-clinical testing, pediatric brain tumors

## Abstract

Corticosteroids, such as dexamethasone, are routinely used as palliative care in neuro-oncology for their anti-inflammatory benefits, however many patients experience dose limiting side effects caused by glucocorticoid response element (GRE)-mediated transcription. The purpose of this study was to use a murine model to investigate a new steroid alternative, vamorolone, which promises to reduce side effects through dissociating GRE-mediated transcription and NF-κB -mediated anti-inflammatory actions. To compare vamorolone to dexamethasone in reducing pro-inflammatory signals *in vitro*, murine glioma cells were treated with dexamethasone, vamorolone or vehicle control. Changes in mRNA expression were assessed using the nanostring inflammatory platform. Furthermore, drug efficacy, post-treatment behavioral activity and side effects were assessed by treating two cohorts of brain tumor bearing mice with dexamethasone, vamorolone, or vehicle control. Our investigation showed that treatment with vamorolone resulted in a reduction of pro-inflammatory signals in tumor cells *in vitro* similar to treatment with dexamethasone. Treatment with vamorolone resulted in a better safety profile in comparison to dexamethasone treatment. Vamorolone- treated mice showed similar or better activity and survival when compared to dexamethasone-treated mice. Our data indicate vamorolone is a potential steroid-sparing alternative for treating patients with brain tumors.

## INTRODUCTION

Survival rates of CNS cancers have increased as a result of advances in surgical and medical treatments and there is increased attention towards minimizing adverse treatment effects and long term sequelae [1]. Edema and intracranial pressure caused by the tumor are major causes of morbidity in patients with brain cancers [2] and are treated with high dose corticosteroids. Dexamethasone is the treatment of choice due to its relatively long half-life, low mineralocorticoid potency, and low Cushing threshold [3, 4]. By decreasing intracranial mass effect, corticosteroids diminish fatigue, headaches, cranial nerve palsies, weakness, ataxia and spasticity, thereby improving a patient's neurological function, activity and comfort [5]. The mechanism of dexamethasone's anti-inflammatory action is believed to be through modulating of expression of vascular endothelial growth factor (VEGF), thus controlling tumor vascularity, and antagonizing prostaglandins secreted by microglia and astrocytes [6]. Glucocorticoid-mediated efficacy has also been attributed to transrepressive mechanisms such as inhibition of NF-κB, a pro-inflammatory transcription factor shown to promote genetic instability and aid in the proliferation and survival of malignant cells, promote angiogenesis and metastasis, subvert adaptive immunity, and alter responses to chemotherapeutic agents [7–10]. NF-κB response is a hallmark of inflammation and has been shown to result in a negative prognosis for patients diagnosed with glioma [11]. IL10, IL6 and TNF are proinflammatory cytokines in the NF-κB pathway, and are released during tissue injury. Furthermore, the role of NF-κB in increasing the expression of pro-inflammatory cytokines including IL10, IL16 and TNF has been previously described in cerebral ischemia [12, 13].

Unfortunately treatment with corticosteroids is associated with severe long-term side effects including a cushingoid appearance which distorts the patient's face and body, generalized edema which can result in splitting of the skin with open sores, immunosuppression, mood change, hyperphagia, insomnia, hypertension, hyperglycemia, and muscle weakness, as well as long term adverse sequelae like osteopenia and stunted growth [14]. These side effects diminish comfort and quality of life and are often dose-limiting. It is widely believed that these glucocorticoid-mediated side effects are attributed to the transactivation sub-activity in which the glucocorticoid receptor/ligand complex binds to glucocorticoid responsive elements (GREs) in the promoter regions of target genes where they affect gene transcription.

ReveraGen BioPharma Inc. (Rockville, MD) has developed a novel anti-inflammatory drug, vamorolone, which is structurally related to glucocorticoids. It has been optimized for retention of transrepressive activities (i.e. NF-κB inhibition) and loss of transactivation activities (GRE-mediated transcriptional activities) [15, 16]. The potent anti-inflammatory action and reduced side effect profile of vamorolone has been demonstrated in in-vitro and in-vivo models of muscular dystrophy[15], allergic lung inflammation [17], multiple sclerosis [18], and inflammatory bowel disease [19].

We tested the effects of vamorolone and dexamethasone *in vitro* and *in vivo* using primary brain tumor cells and murine models. The aims of the study were to evaluate the anti-inflammatory mechanism of action of dexamethasone and vamorolone, *in vitro*, and to compare efficacy and side effects of the two agents in mouse models of brain tumors. We hypothesized that both agents would suppress expression of NF-κB -regulated cytokines *in vitro* and that the agents would have similar efficacy, with vamorolone showing reduced side effects.

## RESULTS

### Vamorolone treatment *in vitro* reduces NF-κB-mediated cytokine expression

To assess the role of vamorolone and dexamethasone on cytokine expression *in vitro*, murine glioma cells [20] were treated with vamorolone, dexamethasone, and DMSO (vehicle control) for 6, 12, 18 and 22 hours, as described in the Methods. mRNA expression profiling revealed reduced levels of all 3 cytokines after 12 hours of treatment with both dexamethasone and vamorolone (Supplementary Figure S1).

### *In vivo* treatment with vamorolone results in reduced glucocorticoid-mediated side-effects when compared to dexamethasone

**Figure 1:**
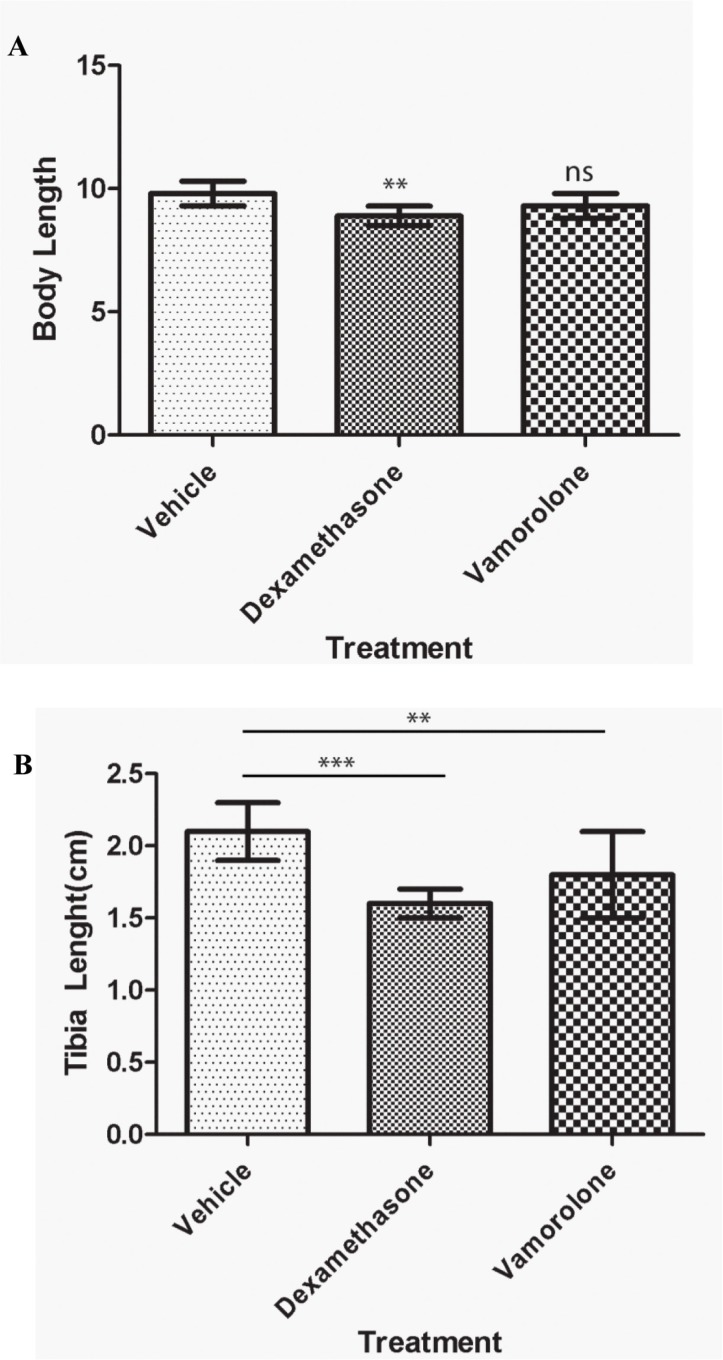
Vamorolone does not inhibit the growth of mice in vivo Tumor bearing mice were treated with vehicle (cherry syrup, control), dexamethasone and vamorolone and mice body length (**A**) and tibia length (**B**) were measured at the endpoint. Dexamethasone treatment significantly reduces the growth of tumor bearing mice as measured at the endpoint (A) Dexamethasone also decreased the bone growth as assessed by tibia measures (B) vamorolone displayed significant measures close to the vehicle treatment. One-way Anova, *p < 0.05, **p < 0.01, ***p < 0.001.

Tumor developing mice were treated with vamorolone, dexamethasone or vehicle control to assess the known side effects of steroids which include stunted growth, reduced bone growth and immunotoxicity. Mice treated with dexamethasone were significantly shorter (8.9 ± 0.4 cm) than mice treated with vehicle control (cherry syrup) (10.1 ± 0.3 cm) (Figure 1A, Supplementary Table S1). However, mice treated with vamorolone did not exhibit the stunted growth, when compared to vehicle- treated and dexamethasone treated mice (Figure 1B). Indeed, tibia length measurements confirmed this finding (Figure 1B). Vehicle-treated mice had tibia lengths of 2.2 ± 0.1 cm, while in comparison, dexamethasone treated mice exhibited significantly shorter tibia length (1.6 ± 0.1 cm, *p* < 0.001). In all, vamorolone treated mice showed tibia length measures similar to the vehicle control treated mice (Figure 1B).

**Figure 2:**
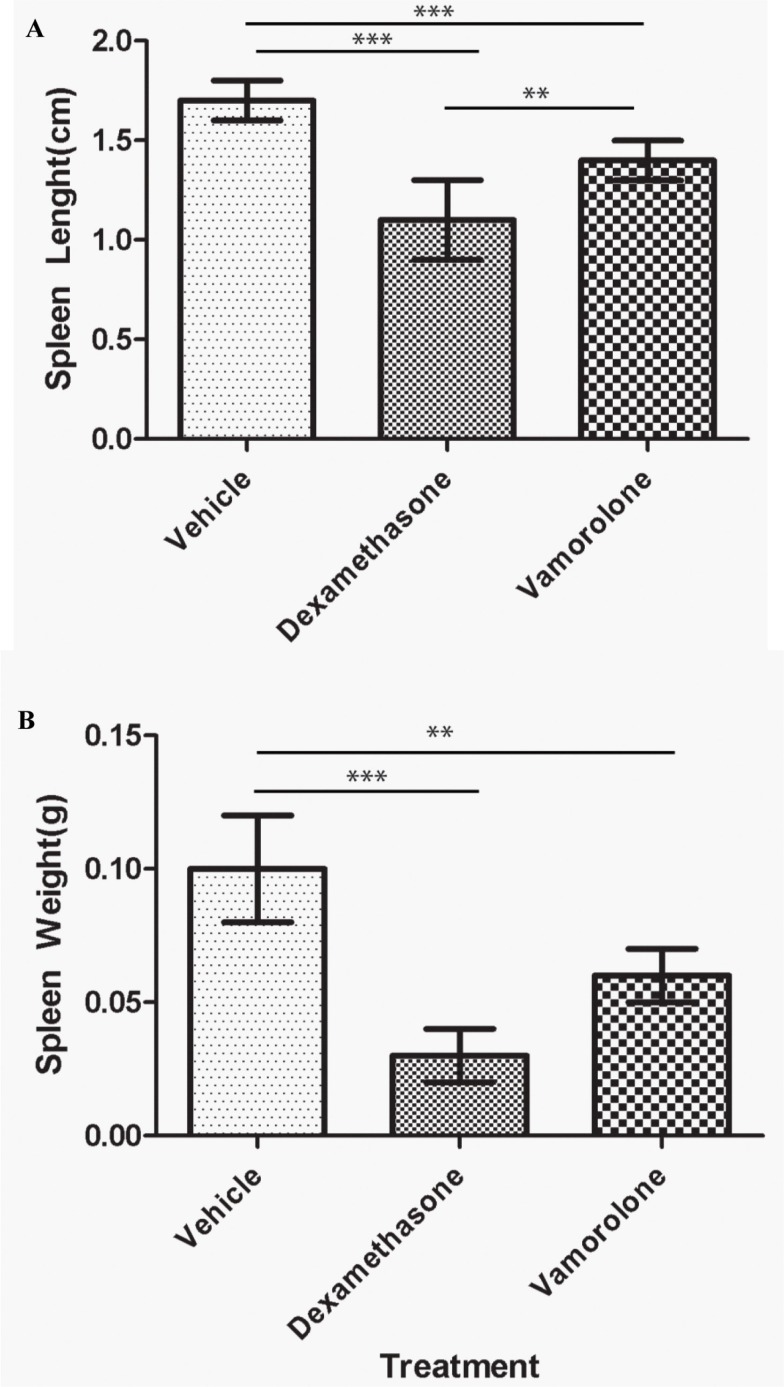
Vamorolone is less immunotoxic compared to dexamethasone Mice were treated with vehicle, dexamethasone or vamorolone and spleen was analyzed for size and weight. Dexamethasone significantly reduced the spleen size (**A**) and weight (**B**) in tumor bearing mice compared to vamorolone or untreated mice. However, mice treated with dexamethasone showed significantly smaller spleen when compared to vamorolone or vehicle- treated mice. One-way Anova, **p < 0.01, ***p < 0.001.

Steroid treatment also results in immunotoxic side effects, further complicating treatment modalities of childhood cancers. To assess the effect of steroid treatment on the systemic immune function *in vivo*, gross examination of the spleen was performed. Treatment with either vamorolone or dexamethasone resulted in significant reduced spleen length (Figure 2A, Supplementary Table S1) and weight (Figure 2B, Supplementary Table S1) while, dexamethasone-treated mice showed significantly smaller spleen sizes (1.1 ± 0.2, *p* < 0.001) and mass (0.03 ± 0.01, *p* = 0.001) when compared to vamorolone.

### Vamorolone increases physical activity of tumor bearing mice

**Figure 3:**
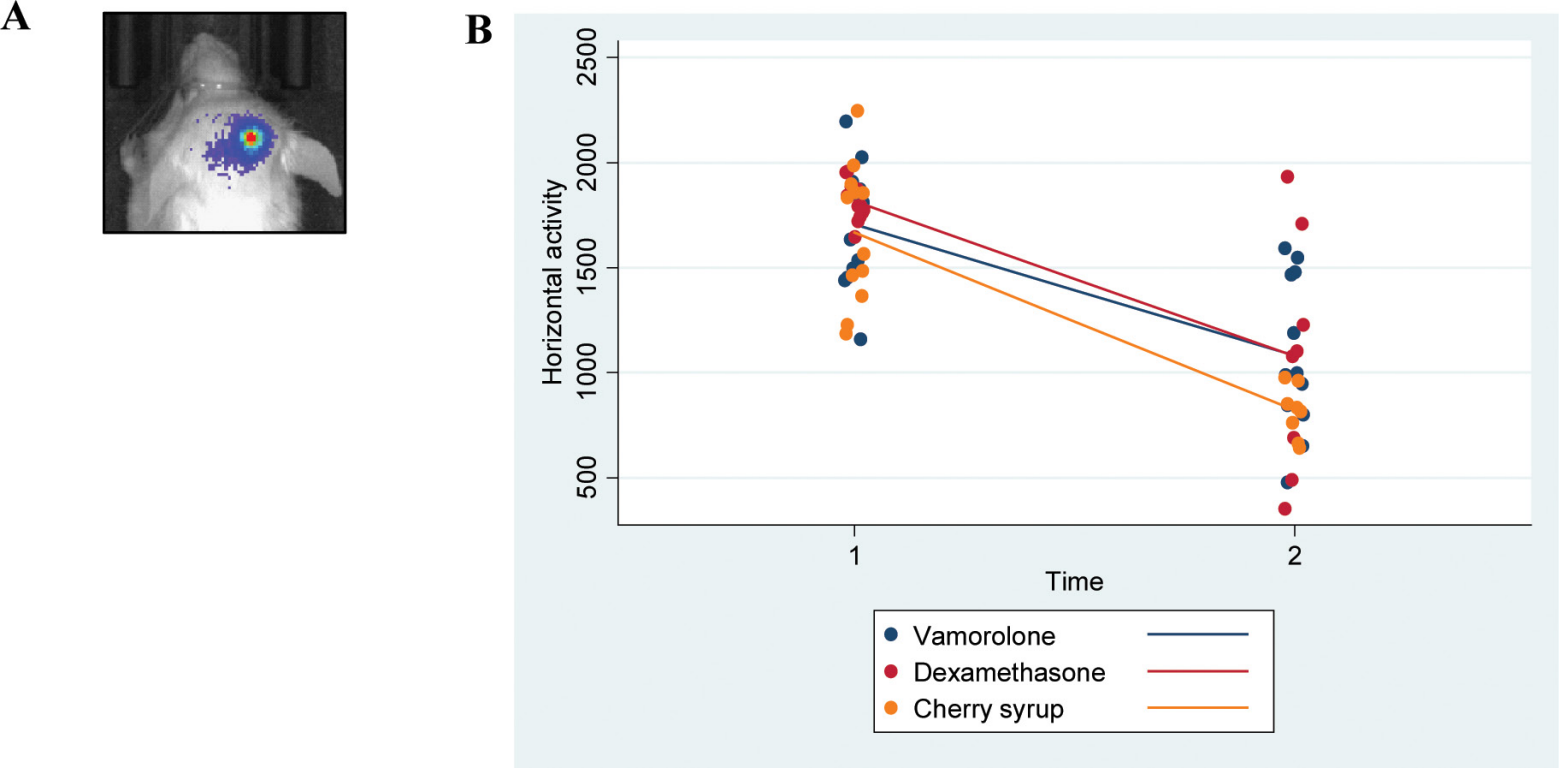
Vamorolone and dexamethasone increase the activity of tumor bearing mice Mice injected with glioma cells (PKC-L) were imaged for signs of tumor formation (**A**) Tumor breading mice were treated with dexamethasone, vamorolone or control vehicle and horizontal activity was measured using VersaMax open field activity monitoring system at both pre and post-treatment time points (**B**) Although a significant decrease in activity was observed from pre-treatment to post-treatment, mice treated with vamorolone showed an increased activity when compared with vehicle-treated mice.

**Figure 4:**
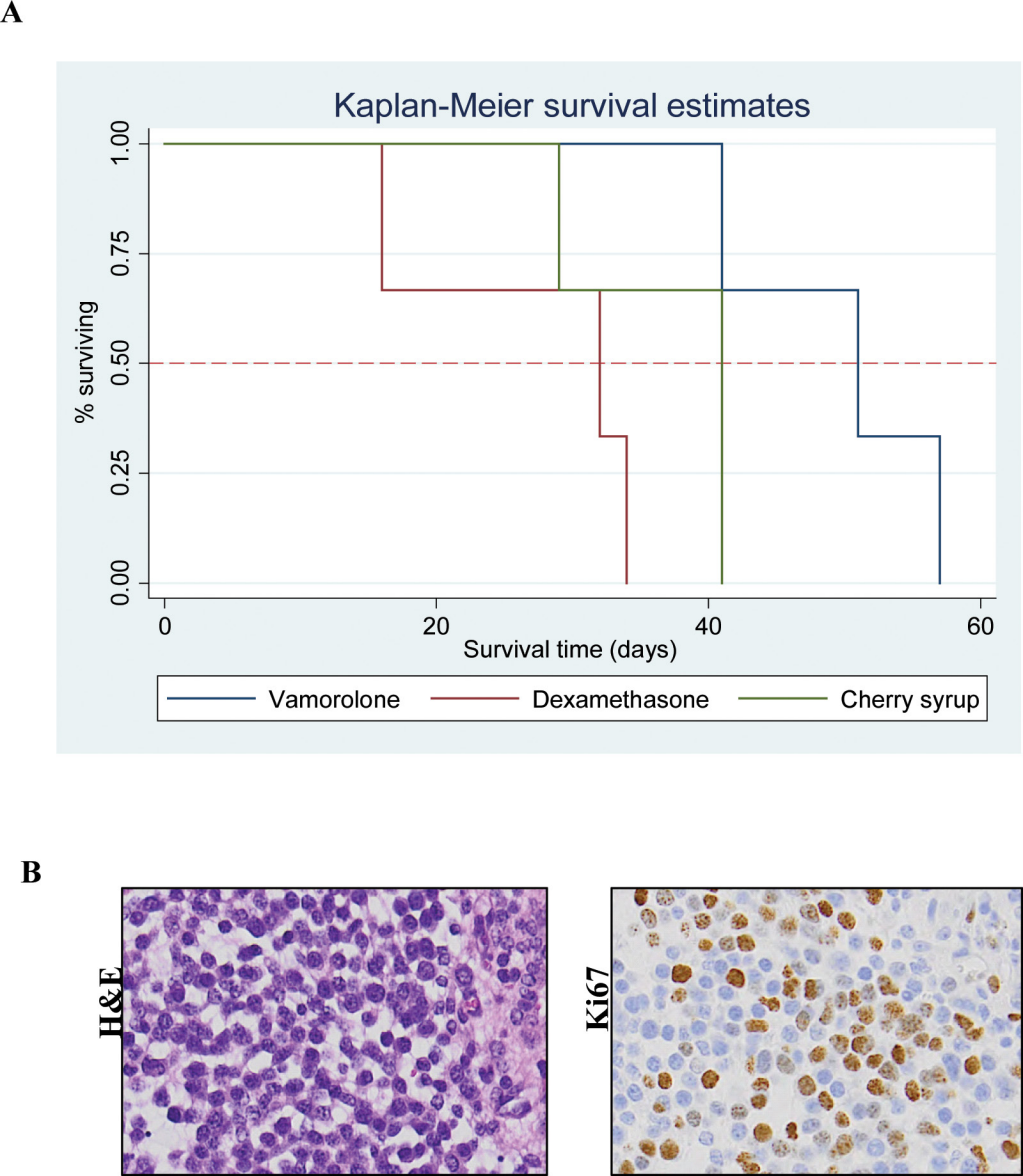
Tumor bearing mice when treated with vamorolone have better survival Tumor bearing mice treated with vamorolone survive longer. Kaplan-Meier plot analysis showed that the vamorolone treated mice show a significantly (*p* = 0.025) increased survival time over those treated with dexamethasone or vehicle control (**A**) Necropsy analysis of brain harvested from these mice showed hypercellularity and proliferative tumors as shown by H&E and Ki67 respectively, 20X magnification (**B**).

Tumor bearing mice (Cohort-2 under Methods, were assessed for behavioral activity using VersaMax open field activity measuring system (Supplementary Table S2). For these experiments, mice were injected with murine glioma PKC-L cells and allowed for tumor formation as assessed by live imaging (Figure 3A). Two weeks post tumor cell injection, mice were treated with dexamethasone, vamorolone and vehicle control for five weeks. All treatment groups showed consistent decreased activity due to tumor formation (Supplementary Table S3). However, both dexamethasone and vamorolone treatment resulted in an increase in activity compared to vehicle-treated tumor bearing mice (Figure 3B). This cohort of mice was further monitored for survival. Our analysis indicated a significantly (*p* = 0.025) increased median survival time of mice treated with vamorolone when compared to mice treated with dexamethasone or vehicle control (Figure 4A). Tumor formation in all mice in this cohort was validated by histological staining of brain at necropsy, indicating the presence of highly infiltrating tumors (Figure 4B).

## DISCUSSION

Development of therapeutics that enhance the palliative care of patients with CNS malignancies is important for effective treatment. Many patients with CNS cancers respond well initially to steroid treatment, used to diminish fatigue, pain, and focal neurologic deficits caused by tumor-related edema and intracranial mass effect [21, 22]. However, the debilitating side effects of steroid treatment frequently counteract the goal of improved quality of life. Adverse side effects may be dose-limiting, with a recent survey by the European Society for Pediatric Oncology (SIOPE) DIPG network reporting a toxicity incidence of 85%. In patients with terminal illness, this tragically eliminates the option of an oral medication to effectively manage pain and fatigue, and increase physical function. Steroid side effects are also associated with long term adverse sequelae in survivors. Therefore, there is a dire need to develop alternative anti-inflammatory drugs that penetrate the CNS and provide steroid efficacy without the severe side effects.

The exact molecular pathways influenced by steroids are not well understood, and clinical trials were never conducted prior to the routine clinical use in pediatric cancer. However previous studies have elucidated the mechanisms of glucocorticoid-mediated efficacy via transrepressive mechanisms such as inhibition of the NF-κB, and led to development of dissociative steroids [15–18]. Vamorolone is a novel steroid analogue that has shown efficacy in inducing an anti-inflammatory response in animal models of asthma, multiple sclerosis, inflammatory bowel disease, and muscular dystrophies, and has shown fewer side effects. We now demonstrate that vamorolone treatment results in reduced inflammatory signals *in vitro* and has efficacy *in vivo* in brain tumor murine model.

In our experiments, vamorolone was capable of reducing NF-κB pathway inflammatory cytokines in mouse primary DIPG cells and we plan on conducting dose response experiments in future studies to assess the drug-target effects. In terms of efficacy, treatment with vamorolone was associated with increases in physical activity, possibly associated with less tumor burden, and there was a 19% increase in survival of mice treated with vamorolone relative to dexamethasone and vehicle control mice. Furthermore, vamorolone showed a reduced side effect profile compared to dexamethasone in brain tumor-bearing mice which grew tumors as pups. Our data therefore indicate vamorolone as a potential suitable steroid alternative for symptoms caused by CNS tumor-related edema.

The study had several limitations: we are unable to determine if the effects seen as a result of the nanostring experiment, which measures mRNA expression, are transcriptional or post protein. Attempts to confirm this data with ELISA resulted in much variation and lacked sensitivity to validate the findings. The precise mechanism of dexamethasone in reducing CNS edema and cellular damage is unknown, with possibilities including working via the inflammasome, i.e. Na^+^ and water movement and intracellular Na^+^/K^+^ normalization, or alternatives to stabilize the blood-brain and blood-nerve barrier. This study was not designed to evaluate these actions *in vitro*. *In vivo* study conclusions are limited by the lack of direct testing of tumor-related edema, although the measurement of edema in patient populations is highly variable and not considered a valid endpoint for clinical trials. An additional potential limitation is that two different glioma mouse models were used for the *in vivo* experiments. We believed that more robust and relevant pediatric toxicity data would be generated from the genetically engineered model in which pups are known to live slightly longer, whereas behavior and survivor findings would be more robust in an experiment performed with the most aggressive mouse model, using adult mice. The study was strengthened by the fact that both *in vitro* experiments were randomized, controlled and blinded.

The demonstration of the anti-inflammatory mechanism and the positive preclinical profile supports the need for further evaluation of this drug to treat neurologic symptoms in primary CNS malignancies. Indeed, our finding that treatment with vamorolone increases survival is an unexpected outcome, which requires further investigation, particularly in diffuse intrinsic pontine glioma (DIPG), the most lethal childhood brain tumor to which most children succumb between 8 to 12 months post diagnosis. Clinical assessment of vamorolone efficacy in palliative treatment of CNS malignancies in routine post-operative care and its potential to extend survival of children with DIPG is warranted.

## MATERIALS AND METHODS

### Murine cells

The pediatric brainstem glioma murine model was chosen because this model is known to cause fast-growing tumors and edema is a major cause of morbidity patients with brainstem glioma and is treated with high dose corticosteroids. Adherent mouse brainstem glioma cells (BSG cells) and PKC (PDGFβ, H3 K27M, P53 loss) cells derived from genetically engineered mouse brainstem glioma model, as previously described [23], were used for *in vitro* and *in vivo* studies respectively. BSG cells were cultured in Dulbecco's Modified Eagle's Medium containing glucose supplemented with 10% fetal bovine serum, penicillin and streptomycin. PKC cells were grown in NeuroCult basal media supplemented with 10% mouse cell proliferation supplement (Stem cell technologies), glutamine, penicillin-streptomycin, heparin (2 μg/μl), rm EGF (20 ng/mL) and rm FGF (1 ng/mL). These cells were transduced with lentivirus to express luciferase, as described previously [20]. The cultures were maintained at 5% CO_2_ at 37°C.

### *In vitro* treatment study assessing mRNA expression profiling of cytokines

The purpose of this experiment was to assess whether vamorolone and dexamethasone alter inflammation caused by brainstem glioma cells, as measured by cytokine expression. IL10, IL6 and TNF were selected for this study because they are proinflammatory cytokines in the NF-κB pathway, released during tissue injury. Murine glioma cells [20] were expanded in culture and treated with Vamorolone, dexamethasone, and DMSO (vehicle control).

Cells were grown in total media, serum starved for 24 hours and then treated with dexamethasone (20 nM), vamorolone (10 μM) and DMSO in serum-free media. Cells were collected 12 hours post treatment. Total RNA was extracted using Qiagen RNeasy kit (Qiagen, CA). RNA concentration and integrity was evaluated by Agilent 2100 bioanalyzer. Sample preparation, hybridization and detection were carried out according to NanoString manufacturer's instructions (NanoString technologies, WA). RNA (100 ng) expression profiling was performed using the nCounter NanoString Mouse inflammatory panel, and data were analyzed using the nSolver software to produce and compare direct counts of mRNA.

### *In vivo* studies

J:Nu and NOD *SCID* gamma mice were used in accordance with our protocol approved by Children's National Health System IACUC (#292-12-05, #30425).

### *In vivo* studies using brainstem glioma model with orthotopic intracranial injections

Experiments assessing dexamethasone and vamorolone efficacy and toxicity were performed on two cohorts of mice who underwent orthotopic injections - P2 pups J:Nu (Cohort-1) for toxicity assessments and 3 week old NOD *SCID* mice (Cohort-2) mice for efficacy assessments. The injection site was sterilized and 300,000 live tumor cells in a total volume of 2 μl were injected into right cortex (2 mm right from midline, 2 mm deep) using a gastight syringe (Hamilton, Reno, NV) with a 26 gauge needle. Mice in Cohort-1 were injected with brainstem glioma cells derived from genetically engineered mouse model as described before [23]. Briefly, the cells were derived from mouse brainstem tumors that were generated by overexpressing PDGFβ in combination with Ink4a-ARF loss in the posterior fossa of neonatal nestin tv-a mice. Mice in Cohort-2 were injected with PKC-L(luciferase) cells from a similar model that had p53 loss and Histone 3 mutation, along with PDGFβ over expression [24]. We have previously shown that mice injected with these cells develop infiltrating cranial tumors [20]. Mice were monitored daily for tumor developing symptoms (like loss of balance, hydrocephalus). For all *in vivo* studies technicians were blinded to treatment conditions [25].

### Toxicity assessment

The experiment performed on Cohort-1 (P2 pups J:Nu) aimed to assess the glucocorticoid-mediated side effect profiles of dexamethasone and vamorolone in mice growing intracranial tumors. Beginning at eight days post injection, mice were orally administered with vamorolone (30 mg/kg), *n* = 11, dexamethasone (2.4 mg/kg) *n* = 7, or vehicle control (cherry syrup 1 mL/kg), *n* = 11, daily, for 15 weeks following guidelines for pre-clinical models [25]. The doses were chosen based on previous studies of vamorolone [15–18] and dexamethasone dose was calculated using the equivalent surface area dosage conversion factor [26]. To assess the toxicity of the steroids, mice (Cohort-1) were sacrificed at the endpoint i.e. when they showed signs of tumor (eg. lethargy, hunched back and ataxia) [20]: spleen, brain and tibia were harvested, weighed (spleen and brain) and measured (spleen and tibia). Brain was fixed in 10% formalin to generate H&E and Ki67 staining to assess the tumor.

### Efficacy assessment

Three week old NOD *SCID* mice (cohort-2) were used to assess survival and behavioral activity Mice were injected with PKC-L tumor cells at three weeks of age and were treated daily with vamorolone (30 mg/kg) *n* = 3, dexamethasone (2.4 mg/kg) *n* = 3, or vehicle control (cherry syrup 1 mL/kg) *n* = 3 two weeks post-injection of PKC-L. Horizontal activity was assessed before and after treatment using the VersaMax open field activity monitoring system (AccuScan Instruments, Inc. Ohio) [27]. In accordance with manufacturer guidelines, mice were acclimated to the activity field chambers a week before collecting data and data was collected for 1 hour for four days to measure horizontal activity (units) and rest time (seconds). Additional measures of movement and rest were obtained at one and four weeks post injection with tumor cells, at the same time every day before 2PM. A survival analysis was also performed on Cohort-2 and the resulting survival curves plotted using the Kaplan Meier method.

### Live animal imaging

Two weeks post injection; live imaging was performed on mice injected with PKC-L cells to assess the tumor formation. Mice received intraperitoneal D- luciferin (5 mg/Kg) and 15 minutes later were anaesthetized with isoflurane. Luciferase was measured using IVIS imaging system according to manufacturer's instructions (Perkin Elmer, MA).

### Statistical analysis

All continuous outcomes were assessed for normality using a combination of the Shapiro-Wilk normality test and visual inspection of histograms. Most measures were analyzed using one-way ANOVA with *post-hoc* pair-wise comparisons and *p*-values were adjusted multiple comparisons using the Sidak method. The consecutive day measures for horizontal activity 3 comparison groups were assessed using a repeated measures analysis of variance (ANOVA) models to test the hypothesis that there is a significant difference in assessments between treatment groups (dexamethasone and vamorolone) and between time points (pre and post treatment with steroids). The analysis used two repeated variables, time and day, to evaluate a difference in assessments by group and by time, and to test for a difference in assessments with day. Survival was assessed using a log-rank test to compare overall survival between the three treatment groups.

## SUPPLEMENTARY MATERIALS FIGURES AND TABLES


