# Supplementary material for: Vamorolone, a dissociative steroidal compound, reduces pro-inflammatory cytokine expression in glioma cells and increases activity and survival in a murine model of cortical tumor
Source: Oncotarget. 2016 Dec 21;8(6):9366–74. doi: 10.18632/oncotarget.14070 (PMC5354737; doi:10.18632/oncotarget.14070)
Supplement: Supplementary file 1 [file oncotarget-08-9366-s001.pdf]

Vamorolone, a dissociative steroidal compound, reduces pro-inflammatory cytokine expression in glioma cells and increases activity and survival in a murine model of cortical tumor

Supplementary Materials

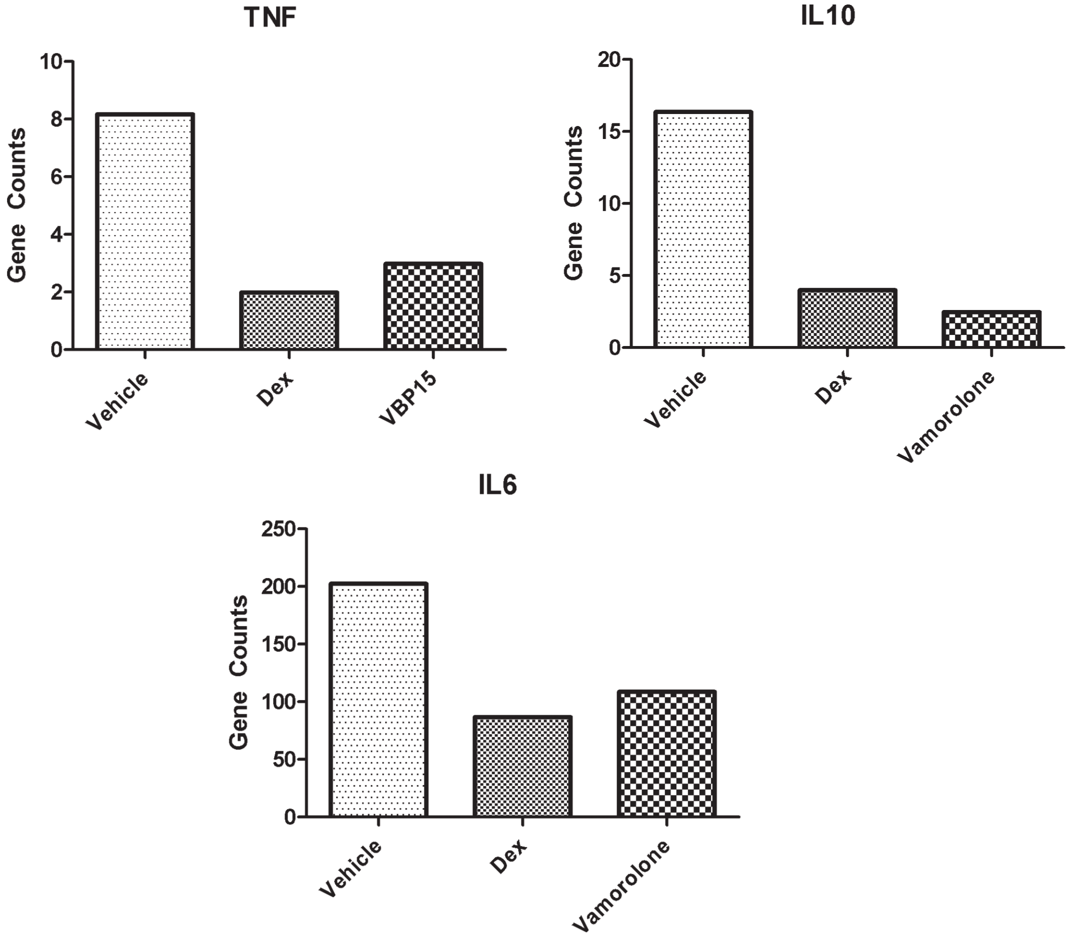

Supplementary Figure S1: Dexamethasone and vamorolone decrease inflammatory cytokines associated with NF-κB pathway *in vitro*. Tumor cells were expanded in culture and treated with vehicle (DMSO, control), dexamethasone (Dex) or vamorolone. mRNA profiling using NanoString mouse inflammatory assay was done using mRNA extracted at 6, 12, 18, and 22 hours of drug exposure. The expression levels of proinflammatory signals including TNF (tope left panel), IL6 (top right panel) and IL10 were decreased compared to the vehicle treated cells.

Supplementary Table S1: Measures of treatment groups to assess the gluco-corticoid mediated side effects in mice

Measure	Vehicle	Dexamethasone	Vamorolone	Overall *p*-value	Significantly different means
	Mean ± SD	Mean ± SD	Mean ± SD		
Body length (cm)	10.1 ± 0.3	8.9 ± 0.4	9.6 ± 0.5	0.0007	Vehicle vs. dex. ($p = 0.001$)<br>Dex. vs. Vamorolone ($p = 0.041$)
Spleen weight (g)	0.11 ± 0.01	0.03 ± 0.01	0.06 ± 0.01	< 0.001	Vehicle vs. dex. ($p < 0.001$)<br>Vehicle vs. Vamorolone ($p < 0.001$)<br>Dex. vs. Vamorolone ($p = 0.001$)
Spleen length (cm)	1.6 ± 0.1	1.1 ± 0.2	1.4 ± 0.1	< 0.001	Vehicle vs. dex. ($p < 0.001$)<br>Vehicle vs. Vamorolone ($p = 0.002$)<br>Dex. vs. Vamorolone ($p < 0.001$)
Tibia length (cm)	2.2 ± 0.1	1.6 ± 0.1	1.9 ± 0.1	< 0.001	Vehicle vs. dex. ($p < 0.001$)<br>Vehicle vs. Vamorolone ($p < 0.001$)<br>Dex. vs. Vamorolone ($p = 0.021$)

All measures were taken at the end point. All measures are found to be normally distributed and one-way ANOVA with post-hoc pair-wise comparisons was performed on these measures and adjustment of p-values for multiple comparisons was done using the Sidak method.

Supplementary Table S2: Schematic to measure the activity using VersaMax open field activity monitoring system

Acclimation:

Step	Mice in the test room	Mice in the test chambers	Mice back into the cage
Time	10 minutes	60 minutes	Until next day

Data Collection:

Step	Mice in the test room	Enter data parameters	Pre beam check	Mice in the test chambers	Mice back into the cage
Time	10 minutes	N/A	N/A	60 minutes	Until next day

Mice were acclimatized to the system one week prior to the activity measurement (top panel). Data was collected for 4 consecutive days (bottom panel). The system collects six readings in one hour (1 reading every 10 minutes).

Supplementary Table S3: Horizontal activity analyses across the groups

Measurement	Comparison groups	Group effect		Measurement Time effect		Day effect	Overall r^2 of model
		p-value	β coef.	*p*-value	β coef.	*p*-value*	
Horizontal activity	Vamorolone vs. Dex	0.61	–99.1	0.019	–442.1	0.25	0.689
	Vamorolone vs. Vehicle	0.52	–550.9	0.012	–442.1	0.030	0.863
	Dex vs. Vehicle	0.16	–664.6	0.06	–668.8	0.88	0.734

Activity measures compared across three groups dexamethasone (Dex), vamorolone and vehicle. Group (vamorolone vs Dex, vamorolone vs vehicle, Dex vs vehicle), Measurement time (pre-treatment to post-treatment), day (four day measurements).
